# Balance and Gait Improvements of Postoperative Rehabilitation in Patients with Parkinson's Disease Treated with Subthalamic Nucleus Deep Brain Stimulation (STN-DBS)

**DOI:** 10.1155/2019/7104071

**Published:** 2019-08-04

**Authors:** Kazunori Sato, Noriaki Aita, Yoshihide Hokari, Eriko Kitahara, Mami Tani, Nana Izawa, Kozo Hatori, Ryota Nakamura, Fuyuko Sasaki, Satoko Sekimoto, Takayuki Jo, Genko Oyama, Taku Hatano, Yasushi Shimo, Hirokazu Iwamuro, Atsushi Umemura, Nobutaka Hattori, Toshiyuki Fujiwara

**Affiliations:** ^1^Department of Rehabilitation Medicine, Juntendo University Hospital, Tokyo, Japan; ^2^Department of Rehabilitation Medicine, Juntendo University Graduate School of Medicine, Tokyo, Japan; ^3^Department of Neurology, Juntendo University Graduate School of Medicine, Tokyo, Japan; ^4^Department of Research and Therapeutics for Movement Disorders, Juntendo University Graduate School of Medicine, Tokyo, Japan; ^5^Department of Neurosurgery, Juntendo University Graduate School of Medicine, Tokyo, Japan

## Abstract

**Background:**

Deep brain stimulation of the subthalamic nucleus (STN-DBS) is a surgical treatment to reduce the “off” state motor symptoms of Parkinson's disease (PD). Postural instability is one of the major impairments, which induces disabilities of activities of daily living (ADLs). The effectiveness of STN-DBS for postural instability is unclear, and the effect of rehabilitation following STN-DBS has remained uncertain.

**Objective:**

The purpose of this study was to examine changes in balance ability, gait function, motor performance, and ADLs following 2 weeks of postoperative rehabilitation in PD patients treated with STN-DBS.

**Methods:**

Sixteen patients were reviewed retrospectively from February 2016 to March 2017. All patients were tested in their “on” medication state for balance and gait performance using the Mini-Balance Evaluation Systems Test (Mini-BESTest) and the Timed “Up and Go” (TUG) test before the operation, after the operation, and during the discharge period. The UPDRS motor score (UPDRS-III) and Barthel Index (BI) were assessed before the operation and during the discharge period. Rehabilitation focused on muscle strengthening with stretching and proactive balance training. Friedman's test and the post hoc Wilcoxon's signed-rank test were used to analyze the balance assessments, and ANOVA and the post hoc Tukey's test were used to analyze gait performance. The significance level was *p* < 0.05.

**Results:**

During the discharge period, the Mini-BESTest and TUG were significantly improved compared with the preoperative and postoperative periods (*p* < 0.05). There were no differences between preoperative and postoperative periods in the Mini-BESTest (*p*=0.12) and TUG (*p*=0.91). The BI and motor sections of the UPDRS did not differ significantly between the preoperative and postoperative periods (*p*=0.45, *p*=0.22).

**Conclusion:**

The results of this study suggest that postoperative rehabilitation improves balance and gait ability in patients with PD treated with STN-DBS.

## 1. Introduction

Deep brain stimulation of the subthalamic nucleus (STN-DBS) has become an effective therapy for advanced Parkinson's disease (PD). STN-DBS reduces motor symptom severity, including the tremor, bradykinesia, rigidity, and dystonia, during the medication “off” state. Patients with advanced PD show postural instability and have an increased risk of falls in daily living [1]. The effects of STN-DBS on posture and balance function are unclear [2–8]. A meta-analysis of nonsurgical PD patients showed a significant effect of physical therapy on balance function as measured by the Timed “Up and Go” (TUG) test, Functional Reach test, and Berg Balance Scale [9]. However, the effectiveness of postoperative rehabilitation with STN-DBS in PD has not been well studied. Only one article has reported significant improvements of the UPDRS motor score and activities of daily living (ADLs) scores (Functional Independence Measure and Barthel Index (BI)) with postoperative rehabilitation in PD patients [10]. They did not assess balance function.

The purpose of this study was to investigate the effects of postoperative rehabilitation on balance and gait function in patients with PD treated with STN-DBS using evaluations that could detect more specific balance and gait dysfunctions.

## 2. Methods

### 2.1. Participants

In this retrospective study, 32 PD patients who underwent STN-DBS in our hospital from February 2016 to March 2017 were recruited. The inclusion criteria were that the patients had received STN-DBS and undergone two-week postoperative physical therapy. The indications for STN-DBS were (i) a good response to levodopa (over 30% improvement on the L-dopa challenge test); (ii) motor complications (dyskinesia, fluctuation); (iii) no dementia or psychiatric problems; and (iv) a precise diagnosis of PD by neurologists specializing in movement disorders. The exclusion criteria of this study were (i) unable to walk independently; (ii) severe complications such as lumbar spondylolisthesis that impaired the patient's balance ability; (iii) postoperative psychiatric problems; (iv) orthostatic hypotension; or (v) lack of clinical data in the medical record.

### 2.2. Therapeutic Exercise

All patients underwent muscle strengthening with stretching and proactive balance training for 40 minutes by experienced physical therapists for approximately 14 days during their hospitalization period (Table 1). Therapeutic exercise consisted of (i) range of motion (ankle, knee, hip, and trunk), (ii) dynamic balance exercise in the quadrupedal and standing positions, and (iii) gait training. The patients underwent modulation of DBS and appropriate medication to achieve the best “on” state.

### 2.3. Clinical Evaluations

Before the surgery (PRE), subjects were tested in all clinical evaluations when they were in the “on” medication state, typically 60–90 minutes after intake of antiparkinsonian medicine. Three days after the implantation of STN-DBS (POST), subjects' balance and gait functions were assessed in the “on” state at the same time as above with antiparkinsonian medicine without stimulation because it was necessary to wait for attenuation of the microlesion effect before starting stimulation. During the discharge period (DISC), typically two weeks after the surgery, the subjects underwent all clinical evaluations with both stimulation and adjusted antiparkinsonian medication that brought about the “on” state. Generally, medications were reduced in the discharge period according to the stimulation.

#### 2.3.1. Mini-Balance Evaluation Systems Test

The balance function of PD patients was assessed with the Mini-Balance Evaluation Systems Test (Mini-BESTest). The Mini-BESTest is a measurement that evaluates balance control and consists of four sections: anticipatory postural adjustments (APA), automatic postural responses (Reactive), sensory integration (Sensory), and dynamic balance during gait (Dynamic gait). This assessment has 14 items with a scale of zero (poor) to two (good), and the maximum score is 28 points [11].

#### 2.3.2. Timed “Up and Go” Test

Gait function was assessed with the TUG test. The TUG test evaluates the time of a movement sequence that involves rising from a chair, walking three meters, turning, returning to the chair, and sitting down on the same chair at a comfortable pace [12]. In addition, the TUG test was assessed with a cognitive task, counting backward by sevens from 100 (TUG-cognitive) [11]. Both the TUG and TUG-cognitive tests are simple but useful tests to assess mobility function and the fall risk of PD patients.

#### 2.3.3. Barthel Index

ADLs assessment was conducted with the BI, which is widely used as the most common ADLs assessment tool. The BI consists of 10 multiple choice items of basic ADLs, with a total scoring range of 0–100 [13]. Higher scores reflect greater physical performance in ADLs.

#### 2.3.4. Unified Parkinson's Disease Rating Scale Motor Score

The Unified Parkinson's Disease Rating Scale (UPDRS) has been widely used as a clinical rating scale for PD [14]. The UPDRS consist of six different sections, and Part 3 (UPDRS-III) reflects the motor performance of PD patients with 14 items (numbers 18 to 31, with a maximum score of 108). Previous studies used numbers 20–26 as cardinal signs (tremor, rigidity, and bradykinesia, with a maximum score of 80), and numbers 29 to 30 as postural instability and gait disability (PIGD) signs (PIGD, with a maximum score of 8) [4]. A higher score reflects the severity of the PD symptoms. In this study, the UPDRS was assessed by a neurologist specializing in movement disorders.

#### 2.3.5. Levodopa Equivalent Daily Dose

According to Tomlinson et al. [15], the levodopa equivalent daily dose (LEDD) in mg was calculated as regular levodopa dose (levodopa × 1), entacapone (levodopa × 0.33), pramipexole (×100), ropinirole (×20), rotigotine (×30), selegiline-oral (×10), rasagiline (×100), amantadine (×1), and apomorphine (×10).

### 2.4. Statistical Analysis

Three periods (PRE, POST, and DISC) of the total Mini-BESTest scores, four subscores (APA, Reactive, Sensory, and Dynamic gait), and LEDD were analyzed with Friedman's test. Wilcoxon's signed-rank test for multiple comparisons was performed as a post hoc test when significant outcomes were found in the primary analyses.

The three periods of the TUG and TUG-cognitive test scores were analyzed with one-way repeated measures analysis of variance (ANOVA). The post hoc Tukey test for multiple comparisons was performed when a significant outcome was found on primary analysis. The UPDRS-III scores and BI scores in the “on” state were compared with Wilcoxon's signed-rank test (PRE and DISC). In all tests, the significance level was *p* < 0.05. All statistical analyses were performed using JSTAT version 2.0. This retrospective study was approved by the institutional ethics review board (JHS 17-0043).

## 3. Results

A total of 32 postoperative cases underwent rehabilitation from February 2016 to March 2017. Sixteen patients were excluded according to the exclusion criteria. One of sixteen patients could not be assessed at PRE because of the dysfunction of gait that resulted from a sudden “off” state. Ten of sixteen patients could not be included because of overlap with another examination or lacking the assessment data in the medical records. Five of sixteen patients could not be assessed due to severe complications (one, orthostatic hypotension; two, lumbar spondylolisthesis; one, knee osteoarthritis; one, severe psychiatric disease). After applying the inclusion and exclusion criteria, 16 patients (5 females and 11 males) remained in this study.  Table 2 shows the demographic data of the 16 included patients and the 16 excluded patients.

### 3.1. Clinical Scale Results

All clinical scale results are presented in Table 3.

### 3.2. Mini-Balance Evaluation Systems Test

Friedman's test showed significant differences among PRE, POST, and DISC (*p* < 0.01) assessments in the total score of the Mini-BESTest. The post hoc Wilcoxon's signed-rank test showed that there were significant differences between the PRE and DISC (*p* < 0.01) assessments and between the POST and DISC (*p* < 0.01) assessments of the total score of the Mini-BESTest, whereas there was no significant difference between PRE and POST (*p*=0.12) assessments in the total score of the Mini-BESTest.

In the four subscores (i.e., APA, Reactive, Sensory, and Dynamic gait) of the Mini-BESTest, Friedman's test showed significant differences among the PRE, POST, and DISC assessments in all subscores. The post hoc Wilcoxon's signed-rank test showed significant differences between PRE and DISC assessments in all subscores except Reactive (*p*=0.065). In the comparison of the POST and DISC assessments, there were significant differences in all subscores. There were no significant differences between PRE and POST assessments in all subscores.

### 3.3. Timed UP and Go Test

One-way repeated measures ANOVA showed significant differences among the PRE, POST, and DISC assessments in the TUG (*F*_2,15_ = 5.95, *p* < 0.01) and TUG-cognitive scores (*F*_2,15_ = 5.32, *p*=0.011). The post hoc Tukey's test showed significant differences between the RE and DISC assessments in the TUG (*p*=0.026) and TUG-cognitive scores (*p*=0.031) and between the POST and DISC assessments in the TUG (*p* < 0.01) and TUG-cognitive scores (*p*=0.016), while there were no differences between the PRE and POST assessments in the TUG (*p*=0.91) and TUG-cognitive scores (*p*=0.96).

### 3.4. UPDRS-III

Wilcoxon's signed-rank test showed no significant differences between the PRE and DISC assessments in the UPDRS-III total score (*p*=0.45), UPDRS-III cardinal score (*p*=0.31), and UPDRS-III PIGD score (*p*=0.49).

### 3.5. Barthel Index

Wilcoxon's signed-rank test showed no significant differences between the PRE and DISC assessments in the BI score (*p*=0.22).

### 3.6. Levodopa Equivalent Daily Dose

Wilcoxon's signed-rank test showed that there were significant differences between the PRE and DISC assessments and between the POST and DISC assessments in the LEDD (*p* < 0.01), while there were no significant differences between the PRE and POST assessments (*p*=0.87). Some patients reduced their antiparkinsonian medication in the POST phase, but most of them maintained their LEDD.

## 4. Discussion

This is the first study to examine the detailed balance and gait abilities of post-STN-DBS surgery PD patients who received postoperative rehabilitation. The present results demonstrated that the postoperative rehabilitation in PD patients treated with STN-DBS was effective in improving balance and gait functions. These findings suggest that the balance and gait functions of PD patients who received rehabilitation treated with STN-DBS could surpass the previous well-medicated balance and gait functions, even though both were in the “on” state.

Many articles reported that the STN-DBS operation was less effective for the axial symptoms of PD patients [2–6]. It was difficult to determine whether the deterioration of axial symptoms was caused by the disease progression itself or the STN-DBS surgery. The present study showed that the STN-DBS operation did not cause deterioration of the axial symptoms. Some authors suggested that postural instability might be induced by the disturbance of the “dopa-responsive” symptoms (such as rigidity, bradykinesia, and tremor) and “nondopaminergic” automatic spinal circuits [16, 17]. Other researchers concluded that STN-DBS was an effective treatment for the “dopa-responsive” motor symptoms but not for the “nondopaminergic” motor symptoms [3]. The balance improvement in the present study might mean that the postoperative rehabilitation in PD patients treated with STN-DBS could have some effect on the “nondopaminergic” motor symptoms.

There has been only one article that reported the effectiveness of postoperative rehabilitation for PD patients treated with STN-DBS [10]. The authors reported that the STN-DBS operation improved the Motor score of UPDRS-III and the ADLs scores (Functional Independence Measure and BI) of PD patients whose Hoehn and Yahr stages were from 2 to 4. In the present study, there were improvements in detailed balance ability and gait function but not in the BI and UPDRS-III. These results might suggest that the BI and UPDRS-III are not appropriate assessment batteries for early detection of balance deficits. In the present study, the included patients were relatively early-stage patients whose Hoehn and Yahr stages were from 2 to 3, and the aim of the operation was the reduction of medication, motor complications, and duration of the “off” state. The “on” state ADLs scores of patients were comparably good even before the operation. Although the ADLs and UPDRS-III scores of the present patients were not significantly different between baseline and after rehabilitation, PD patients showed balance deficits at baseline and showed improvements in the balance scores and gait performance during the discharge period. This might indicate that the Mini-BESTest could detect early balance deterioration in PD patients, and postoperative rehabilitation in PD patients treated with STN-DBS could maximize the balance ability of mild PD patients.

## 5. Limitations

One limitation of this study is that this was a retrospective study with no control group. Because of the ethical constraints, the authors could not intentionally have control patients who did not receive postoperative rehabilitation after the STN-DBS operation. In addition, because of the study design, this study could not evaluate the isolated effect of postoperative rehabilitation and STN-DBS. A further study should plan specific training programs with a dose-matched control study. Moreover, the precise duration of the “on” state was not compared before and after the operation. This study only showed the reduction of LEDD as a benefit of the STN-DBS itself. To solve this issue, the assessment of “on” phase duration should be included in a future study.

## 6. Conclusion

In summary, the results of a retrospective study that assessed the effectiveness of rehabilitation in PD patients treated with STN-DBS were presented. This study appears to demonstrate that the operation itself did not aggravate the postural instability of PD patients, and postoperative rehabilitation with stimulation improved balance ability and gait performance in PD patients.

## Figures and Tables

**Table 1:** Proactive balance muscle strengthening.

Preparation	Active assistive range of motion exercise for ankle, hip, and trunk joints
Dynamic balance exercise	Quadrupedal balance (cat and dog, diagonal balancing exercise)
	Standing balance (toe-heel weight bearing, one-leg standing, step position)
Gait exercise	Active assistive gait training

**Table 2:** Demographic data of 32 PD patients.

	Included (*n*=16)	Excluded (*n*=16)
Age, years, median (IQR)	61.5 (9.5)	65.5 (11.5)
Sex, females, *n* (%)	5 (31)	11 (68)
Duration of disease, years, median (IQR)	13.0 (8.0)	13.5 (4.3)
Duration of medication, years, median (IQR)	11.5 (7.0)	11.0 (4.5)
Hoehn and Yahr stage, median (IQR)	3.0 (1.0)	3.0 (0.3)
Final stimulation setting, median (IQR)		
Pulse, microseconds	60.0 (0.00)	60.0 (7.5)
Hz	130.0 (0.0)	130.0 (15.0)
Volts	1.68 (1.21)	2.00 (0.95)
First LEDD	1216 (614)	1281 (473)
Final LEDD	555 (315)	713 (334)
Dominant affected side, right (%)	12 (75)	9 (56)

IQR, interquartile range; LEDD, levodopa equivalent daily dose.

**Table 3:** The effects of operation and rehabilitation with stimulation.

	PRE	POST	DISC	*p* value Friedman test		*p* value (PRE-POST)	*p* value (POST-DISC)	*p* value (PRE-DISC)
*Mini-BESTTest, median (IQR)*								
Total score	19.0 (5.75)	19.0 (5.5)	23.1 (5.5)	<0.01^*∗∗*^		0.12	<0.01^*∗∗*^	<0.01^*∗∗*^
Subscore, APA	4.0 (1.75)	4.0 (1.75)	5.0 (1.0)	<0.01^*∗∗*^		0.84	<0.01^*∗∗*^	<0.01^*∗∗*^
Subscore, reactive	2.5 (4.5)	2.0 (2.75)	4.0 (3.5)	0.038^*∗*^		0.52	0.017^*∗*^	0.065
Subscore, sensory	4.5 (2.5)	5.0 (2.75)	6.0 (1.0)	0.035^*∗*^		0.64	0.042^*∗*^	<0.01^*∗∗*^
Subscore, dynamic gait	8.0 (1.0)	9.0 (2.0)	9.0 (1.75)	0.011^*∗*^		0.84	0.031^*∗*^	0.016^*∗*^
LEDD (mg), median (IQR)	1216 (614)	1216 (508)	555 (315)	<0.01^*∗∗*^		0.87	<0.01^*∗∗*^	<0.01^*∗∗*^

	PRE	POST	DISC	*p* value ANOVA	*F*	*p* value (PRE-POST)	*p* value (POST-DISC)	*p* value (PRE-DISC)

TUG (seconds), mean (SD)	9.8 (3.9)	10.1 (4.2)	8.1 (2.3)	<0.01^*∗∗*^	5.95	0.91	<0.01^*∗∗*^	0.026^*∗*^
TUG-cognitive (seconds), mean (SD)	16.2 (7.3)	16.6 (11.9)	11.9 (6.1)	0.011^*∗*^	5.32	0.96	0.016^*∗*^	0.031^*∗*^

	PRE		DISC			*p* value Wilcoxon signed-rank test		

UPDRS-III	17.5 (7.75)		13.5 (9.75)			0.45		
UPDRS-III cardinal score	10 (3.5)		7.5 (7.75)			0.31		
UPDRS-III PIGD score	2 (2.5)		1.5 (2.75)			0.49		
BI, median (IQR)	82.5 (17.5)		90.0 (25.0)			0.22		

IQR, interquartile range; SD, standard deviation; Mini-BESTest, Mini-Balance Evaluation Systems Test; PRE, preoperation; POST, postoperation; DISC, discharge; LEDD, levodopa equivalent daily dose; TUG, Timed Up and Go test; UPDRS-III, unified Parkinson's disease rating scale motor score; UPDRS-III cardinal score, unified Parkinson's disease rating scale motor score-cardinal score (20–26); UPDRS-III PIGD score, unified Parkinson's disease rating scale motor score-postural instability and gait disability score (29-30); BI, Barthel Index. ^*∗*^ and ^*∗∗*^ are *p* < 0.05 and *p* < 0.01 for intergroup comparisons.

## Data Availability

The numeric data used to support the findings of this study are included within the article.
